# Single-cell transcriptomic and T cell antigen receptor analysis of human cytomegalovirus (hCMV)-specific memory T cells reveals effectors and pre-effectors of CD8^+^- and CD4^+^-cytotoxic T cells

**DOI:** 10.1111/imm.13783

**Published:** 2024-03-19

**Authors:** Raunak Kar, Somdeb Chattopadhyay, Anjali Sharma, Kirti Sharma, Shreya Sinha, Gopalakrishnan Aneeshkumar Arimbasseri, Veena S. Patil

**Affiliations:** 1 Immunogenomics Lab, National Institute of Immunology, New Delhi, Delhi, India; 2 Department of Transfusion Medicine and Blood Bank, Vardhman Mahavir Medical College and Safdarjung Hospital, New Delhi, Delhi, India; 3 Molecular Genetics Lab, National Institute of Immunology, New Delhi, Delhi, India

**Keywords:** antigen-specific T cells, CD4-CTLs, CD8-CTLs, hCMV, single-cell RNA-Seq, single-cell TCR-Seq, TCR, T_REGs_

## Abstract

Latent human cytomegalovirus (hCMV) infection can pose a serious threat of reactivation and disease occurrence in immune-compromised individuals. Although T cells are at the core of the protective immune response to hCMV infection, a detailed characterization of different T cell subsets involved in hCMV immunity is lacking. Here, in an unbiased manner, we characterized over 8000 hCMV-reactive peripheral memory T cells isolated from seropositive human donors, at a single-cell resolution by analysing their single-cell transcriptomes paired with the T cell antigen receptor (TCR) repertoires. The hCMV-reactive T cells were highly heterogeneous and consisted of different developmental and functional memory T cell subsets such as, long-term memory precursors and effectors, T helper-17, T regulatory cells (T_REGs_) and cytotoxic T lymphocytes (CTLs) of both CD4 and CD8 origin. The hCMV-specific T_REGs_, in addition to being enriched for molecules known for their suppressive functions, showed enrichment for the interferon response signature gene sets. The hCMV-specific CTLs were of two types, the pre-effector- and effector-like. The co-clustering of hCMV-specific CD4-CTLs and CD8-CTLs in both pre-effector as well as effector clusters suggest shared transcriptomic signatures between them. The huge TCR clonal expansion of cytotoxic clusters suggests a dominant role in the protective immune response to CMV. The study uncovers the heterogeneity in the hCMV-specific memory T cells revealing many functional subsets with potential implications in better understanding of hCMV-specific T cell immunity. The data presented can serve as a knowledge base for designing vaccines and therapeutics.

## Introduction

The acquisition of immunological memory to infections is the hallmark of protective immune response. During this conserved process of T cell immunological memory development, the naive T cells that have not previously encountered antigen, differentiate during the primary infection, into memory T cells that have specialized functions in immune defence to a subsequent infection with the same pathogen. A small number of antigen-specific memory T cells can have an enormous impact in directing or misdirecting an immune response upon secondary infection. However, the rarity of antigen-specific memory T cells has limited the detailed characterization of these cells until very recently. The utilization of single-cell genomics to dissect the heterogeneity and understand the global gene expression patterns of these rare antigen-specific memory T cells has advanced our understanding of various disease-specific T cell memory repertoire in an unbiased manner [1–7].

CD4^+^ memory T cells are classified based on their functional properties and in addition, more recently also based on their transcriptomes, into multiple subsets such as but not limited to, T helper (T_H_)1, T_H_2, T_H_17, T_H_1/17, T follicular helper (T_FH_) and T regulatory (T_REG_) cells [8–12]. For example, T_FH_ cells are the specialized providers of help to B cells, and T_REGs_, the regulators of the immune response with their immunosuppressive properties [11, 13, 14]. Furthermore, each of those subtypes can exhibit great functional diversity and plasticity [12]. The peripheral CD4^+^ as well as CD8^+^ memory T cells can also be classified based on their developmental stages as long-lived precursor memory and short-lived effector memory T cells, such as stem cell memory T cells (T_SCM_), central memory T cells (T_CM_), effector memory T cells (T_EM_), effector memory T cells expressing CD45RA (T_EMRA_) [15]. The CD8^+^ T cells are known for their cytotoxic functions, and the CD4^+^ T cells, predominantly, for their helper function, where they provide help to other immune cell types, including the CD8^+^ T cells, in resolving the infection. However, others and we have shown that CD4^+^ T cells can also function as cytotoxic T cells and are enriched in the CD4-T_EMRA_ compartment and show marked T cell antigen receptor (TCR) clonal expansion similar to the CD8-T_EMRA_ cells in many acute and chronic viral infections including the cytomegalovirus (CMV) [1, 3, 4, 6, 16–22].

Following initial infection, the human CMV (hCMV) establishes persistence or latency in immune-competent individuals, although intermittent viral shedding can occur [23]. In the absence of an effective immune response especially in immune-compromised individuals, the virus can reactivate and cause the disease. Hence, reactivation of hCMV poses a higher risk for individuals undergoing transplant or chemotherapy, immune-compromised human immunodeficiency virus (HIV) infected individuals, and new-borns of hCMV seropositive mothers [23]. Both CD4^+^ and CD8^+^ T cells have been shown to play important roles in the immune response to CMV [24, 25]. While hCMV-specific CD8^+^ T cells have extensively been studied, studies on CD4^+^ T cells are sparse [2]. Considering that the emergence of the hCMV-specific CD4^+^ T cells precedes that of CD8^+^ T cells, they were mostly thought to perform supportive roles by enhancing the CD8^+^ T cell responses to the hCMV infection. However, subsequent studies have demonstrated additional roles of CD4^+^ T cells, other than the supportive one towards CD8^+^ T cells, in CMV immunity (reviewed in reference [2]). The poorer or lack of hCMV-specific CD4^+^ T cell responses has been associated with persistent viral shedding in urine and saliva as well as severe disease outcomes [24–27]. In individuals undergoing stem cell or organ transplantation, hCMV-specific CD4^+^ and CD8^+^ T cells have been shown to abrogate reactivation [25–29]. Further, during the primary infection, the role of CD4^+^ T cells has been established in resolution of the disease symptoms [30, 31]. The identification of hCMV-specific T cells has classically relied on the measurement of intracellular cytokine production, predominantly interferon-gamma (IFNγ) and to a lesser extent interleukin 10 (IL10), in response to stimulation with peptides derived from various hCMV ORFs (open reading frames) or total viral lysate [2, 6, 16, 32–35]. Several studies, in healthy seropositive individuals, have identified a higher percentage of hCMV-specific T cells, reaching almost as high as ~5%–10% of total CD4^+^ and CD8^+^ T cells for epitopes derived from pp65 and IE-1 proteins of hCMV, with majority of them showing terminally differentiated effector memory phenotype [16, 33, 34]. In addition to producing IFNγ, the hCMV-reactive CD4^+^ T cells have been shown to make cytolytic molecules such as Granzymes, Perforin, and CD107a (degranulation marker), and showed efficient lysis of hCMV-infected dendritic cells and controlled virus dissemination in vitro, suggesting prominent role of cytotoxic CD4^+^ T cells in hCMV immunity [16–20]. CD4^+^ T cells expressing CD25 and FOXP3 (T_REGs_), producing IL10, an immunosuppressive cytokine, have also been observed and are implicated in immune suppression in CMV infection and reactivation [36–39]. However, a detailed characterization of the spectrum of hCMV-specific CD4^+^ memory T cells with respect to their heterogeneity and molecular signatures has been lacking. Hence, the identification and characterization of the overall hCMV-specific memory T cells with respect to their molecular signatures, in hCMV seropositive individuals who are otherwise healthy, will enable the identification of correlates of protective immune response to hCMV.

Hence, in this study, to isolate the spectrum of hCMV-specific T cells, independent of their cytokine profile and human leukocyte antigen (HLA) restriction or not limited to a single peptide (peptide–major histocompatibility complex (MHC) multimers), we used ARTE (antigen-reactive T cell enrichment) assay [40, 41]. The ARTE assay utilizes the surface expression of co-stimulatory molecules (CD154 and CD137) upon TCR engagement with cognate peptide–MHC complex [40, 41]. To dissect the heterogeneity across hCMV-specific T cell memory subsets and TCR clonal diversity, we performed single-cell transcriptomics analysis of over 8000 hCMV-specific memory T cells, isolated based on the surface expression of CD154^+^ and/or CD137^+^ across 10 seropositive donors, after stimulation with pool of hCMV peptides derived from T cell immuno-dominant epitopes of pp65 and IE-1 proteins. Unbiased clustering based on single-cell transcriptomes identified nine distinct clusters of various functional subsets, including T_REGs_, T_H_17, T_H_1 and T_H_1/17, and cytotoxic CD4^+^ T cells (CD4-CTLs). The hCMV-antigen-specific T_REGs_ were enriched for molecules linked to their suppressive function and interferon response genes (T_REG-IFN_). The transcriptomics analysis combined with TCR repertoire analysis revealed that the cytotoxic cells are predominantly of two types: pre-effectors and effectors and are highly clonally expanded. Interestingly, we observed that the transcriptomes of pre-effectors and effectors of CD4-CTLs and CD8-CTLs were largely indistinguishable. Taken together, in an unbiased approach, our study has identified many previously known as well as novel subsets of hCMV-specific memory T cells and their unique transcriptomic signatures, which potentially have implications for understanding the reactivation of hCMV in immune-compromised individuals. This knowledge base can be an important resource for designing vaccines and therapeutics.

## Materials and Methods

### Study subjects

The study subjects were healthy adult donors who donated blood at the Safdarjung Blood Bank during 2019–2020. The donors consented to the use of the plasma and buffy coat samples for research. Donors were HIV-negative and had no history of Hepatitis C infection. The median age was 30.5 years (ranged from 24 to 39 years) and all these donors were male. Approval for the use of this material for research was obtained from the institutional human ethics committees from both the National Institute of Immunology, New Delhi and Vardhman Mahavir Medical College (VMMC) and Safdarjung Hospital, New Delhi, India.

### Peripheral blood mononuclear cell processing

Peripheral blood mononuclear cells (PBMCs) were isolated from buffy coat samples from healthy blood donors obtained from Safdarjung hospital blood bank by density gradient centrifugation using Ficoll-Paque Premium (GE Healthcare Biosciences). PBMCs were cryopreserved in 90% fetal bovine serum (FBS) supplemented with 10% di-methyl sulfoxide (DMSO).

### CMV IgG analysis

ELISA (enzyme-linked immunosorbent assay) was performed on the plasma samples from the donors obtained from the blood bank using the Human Anti-Cytomegalovirus IgG ELISA Kit (Abcam, #ab108724) according to the manufacturer’s protocol to check for the donors’ sero-status. Donors with absorbance values at least 10% higher than that of the cutoff control supplied in the kit, were considered positive. Based on this criterion we found 124 out of 126 donors in our cohort were hCMV IgG^+^ (98.4% positivity) (Figure S1a).

### PBMC stimulation

To analyze or isolate antigen-specific T cells, the ARTE assay was employed with few minor modifications [40, 41]. The hCMV-specific PepTivators (Miltenyi Biotec) derived from pp65 (UL83) and IE-1 (UL123) were used to stimulate PBMCs from CMV-seropositive donors, according to the manufacturer’s protocol. Briefly, PBMCs were thawed and treated with 50 U/mL Benzonase (Sigma) in RPMI before resting overnight in serum-free TexMACS medium (Miltenyi Biotec) supplemented with 1% Penicillin/Streptomycin at 37°C in a 96-well plate. Cells were stimulated for 6 or 24 h at a density of 1 million/100 μL/well of 96-well plates, by the addition of hCMV peptide pools (pp65 and IE-1 PepTivators) at a final concentration of 1 μg/mL in the presence of a blocking CD40 antibody (1 μg/mL; Miltenyi Biotec) and CD28 antibody (1 μg/mL; eBioscience) [1, 40, 41]. Stimulation with αCD3/CD28 DynaBeads Human T cell activator (Invitrogen) (cell to bead ratio of 1:1) served as positive control, while water (vehicle control) served as unstimulated negative control (Figure S1c). The stimulated cells were washed and stained with a cocktail of fluorescent antibodies (Table S2) and analyzed by flow cytometry or processed for single-cell RNA-sequencing using 10X genomics as follows. The hCMV-peptide pool stimulated cells from each donor were washed and individually stained with Cell-hashtag (HTO:Hash Tag Oligo) Total-Seq C antibody (0.5 μg/condition; BioLegend; Table S2) as per the manufacturer’s recommendations for 30 min on ice, and washed two times with MACS buffer (PBS + 2% FBS + 2 mM EDTA). HTO-stained PBMCs from all 10 donors were then pooled and stained with oligotagged TotalSeq C antibody for cell surface proteins for T cell memory markers (CD45RA, CCR7, CD95 and IL7Rα) (0.5 μg/condition; BioLegend; Table S2) for 30 mins on ice. Following washing, the pooled cells were further stained with fluorescence-conjugated surface antibodies to enable FACS sorting of antigen-specific cells (Table S2). Cells were directly FACS sorted without an intermediate MACS column enrichment step using FAC-SAria Fusion Cell Sorter (Becton Dickinson) in LoBind 1.5 mL microcentrifuge (Eppendorf) tubes with 1:1 FBS:PBS supplemented with recombinant RNase inhibitor (1:100, Takara). The live singlet gated CD3^+^ T cells were further gated as per the gating strategy shown in Figure S1d. List of antibodies and stimulation reagents used in the study are provided in Table S2. The flow cytometry data was analyzed using FlowJo software (v10.8).

### Single-cell RNA-Seq

The sorted antigen-specific T cells from various memory compartments were pooled in desired ratios and used for single-cell RNA-Seq and TCR-Seq assay using 10X Genomics kit, 5′ V(D)J with gene expression and cell surface protein expression v1.1 as per the manufacturer’s recommendations. Initial amplification of cDNA libraries and final libraries were performed for 15 and 14 cycles, respectively, for gene expression library. V(D)J and cell surface protein libraries were generated corresponding to each 5′ gene expression library using nine cycles of amplification. Libraries were sequenced on the Illumina NovaSeq6000 sequencing platform for paired-end (PE) 150 reads.

### Single-cell RNA-Seq analysis

Sequenced reads from the 5′ single-cell gene expression and associated V(D)J library were mapped to the GRCh38 reference genome using 10X Genomics’ cellranger multi (v6.1.1) pipeline. The cell surface protein quantification was run as part of the same pipeline. The Seurat package (v4.3) in R (v4.2.2) was used to perform all further downstream analyses [42].

For downstream analysis, only cells that passed the quality control (QC) parameters were retained; cells with UMI (Unique Molecular Identifier) counts >500, number of genes >350 and percent mitochondrial genes <15% were considered as good quality cells. Further, cells were assigned as doublets, singlets or negatives based on the HTO counts using MULTIseqDemux with default parameters in Seurat package (v4.3) [43]. Singlets were assigned to their respective donor based on HTO count. Doublets were removed from downstream analysis. After applying all the QC parameters, we retained 6899 out of 8062 cells for downstream analysis (85.57% of cells passed the QC parameters). Cells from Experiment 1 (3 donors) and Experiment 2 (10 donors) were combined using the Seurat package after applying the QC (Table S1).

For each cell, normalized UMI counts for each gene were obtained by dividing the raw counts by the total counts across all genes. This was multiplied by a scaling factor of 10000 and log-transformed to yield the final normalized values. Prior to clustering, we filtered genes by selecting the top 2000 with the highest variance across cells, using *FindVariableFeatures* with *selection.method*=“*vst*.” These data were then centered and scaled using *ScaleData*, and the top 50 principal components (PCs) were calculated. The top 18 PCs were selected using *ElbowPlot* and *JackStrawPlot* for downstream processing. This was used to build a shared nearest neighbour graph using *FindNeighbors*. The cells were clustered using *FindClusters*, with *algorithm* = 3 and *resolution* = 0.2, determined using the *clustree* function. For visualization and clustering, a 2D UMAP projection of the top 18 PCs was obtained using the *RunUMAP* function, with *metric*=“*euclidean*” [42].

The cell surface protein tag data was normalized using the *NormalizeData* function in Seurat with *normalization.method*=“CLR.” Briefly, for each cell, the hashtag counts were divided by the geometric mean of counts of all unique hashtags prior to log transformation.

### Differential gene expression analysis

We identified differentially expressed (DE) genes using the MAST algorithm integrated within Seurat’s *FindAll-Markers* function using default parameters on log_2_(CPM + 1) counts [42]. Genes in a cluster were reported as DE compared to every other cluster if their log_2_ fold change of expression was >0.25 and BH-adjusted *p* value was <0.05.

### Gene module signature scores and gene set enrichment analysis

Gene module scores were computed using the *AddModuleScore* function in Seurat, with default parameters [42]. Briefly, for each cell, the module score is defined by the mean of the signature gene list (test set) after subtracting the mean expression of an aggregate of control gene lists. Control gene lists (same number as test gene list) are sampled from bins created based on the level of expression of the signature gene list. Gene set enrichment analysis (GSEA) was performed using Qlucore Omics Explorer 3.8 software package [44]. List of the genes used is provided in Table S3.

### Trajectory inference

Single-cell trajectories were computed using the slingshot algorithm within the dynverse/dyno (v0.1.2) suite of trajectory inference pipelines [45]. Raw and normalized UMI counts from cells of clusters 0, 2, 3, 4 and 7 were combined using the dynverse’s wrap_expressions function. Cells were assigned groups by cluster and the number of expected start and end states were set to 1 using *add_prior_information*. Trajectory inference was run using *infer_trajectories* with default parameters, with method = ti_slingshot(). We did not specify any start or end cells for the trajectory.

### Pathway enrichment analysis

DE genes between clusters 2 and 4 were fed to Ingenuity Pathway Analysis software from Qiagen. Enrichment pathways were sourced from Ingenuity core enrichment pathways.

### TCR analysis

For each cell, all information about clonality, expressed TCR chains, their sequences and corresponding UMI counts were obtained as part of the standard 10X Genomics’ cellranger multi pipeline (Table S5).

### Assigning CD4 and CD8 T cells

For cells in clusters 2 and 4, we assigned cells as CD4^+^ T cells, or CD8^+^ T cells, first based on the *CD4* and *CD8A*/*CD8B* transcript counts. Cells with >0 counts for *CD4* were assigned as CD4^+^ T cells (333 cells). For CD8^+^ T cells, we considered the cell as CD8^+^ T cell (735 cells) if the sum of all three CD8 transcripts (*CD8A, CD8B* and *CD8B2*) was >0 and *CD4* transcripts were undetectable. Cells (556) which could not be assigned as either CD4 or CD8 T cell based on this criterion, were further assigned as CD4^+^ (274) and CD8^+^ (12) T cells if they shared the clonotypes with an already assigned CD4^+^ or CD8^+^ T cell. Cells that could not be assigned to either CD4 or CD8 category based on either of these criteria, were called as NA (not assigned) cells (294). Based on these criteria, we had a total of 328 CD4^+^ T cells, 536 CD8^+^ T cells and 149 NA cells in cluster 2 (total 1013 cells) and 279 CD4^+^ T cells, 199 CD8^+^ T cells and 145 NA cells in cluster 4 (total 623 cells).

### Statistical tests

All the statistical tests were done using GraphPad Prism v9.0. Mann–Whitney *U* test was used to perform pairwise comparison and *p* value <0.05 was considered significant.

## Results

### Single-cell transcriptomic analysis of CMV-specific memory T cells

**Figure 1:**
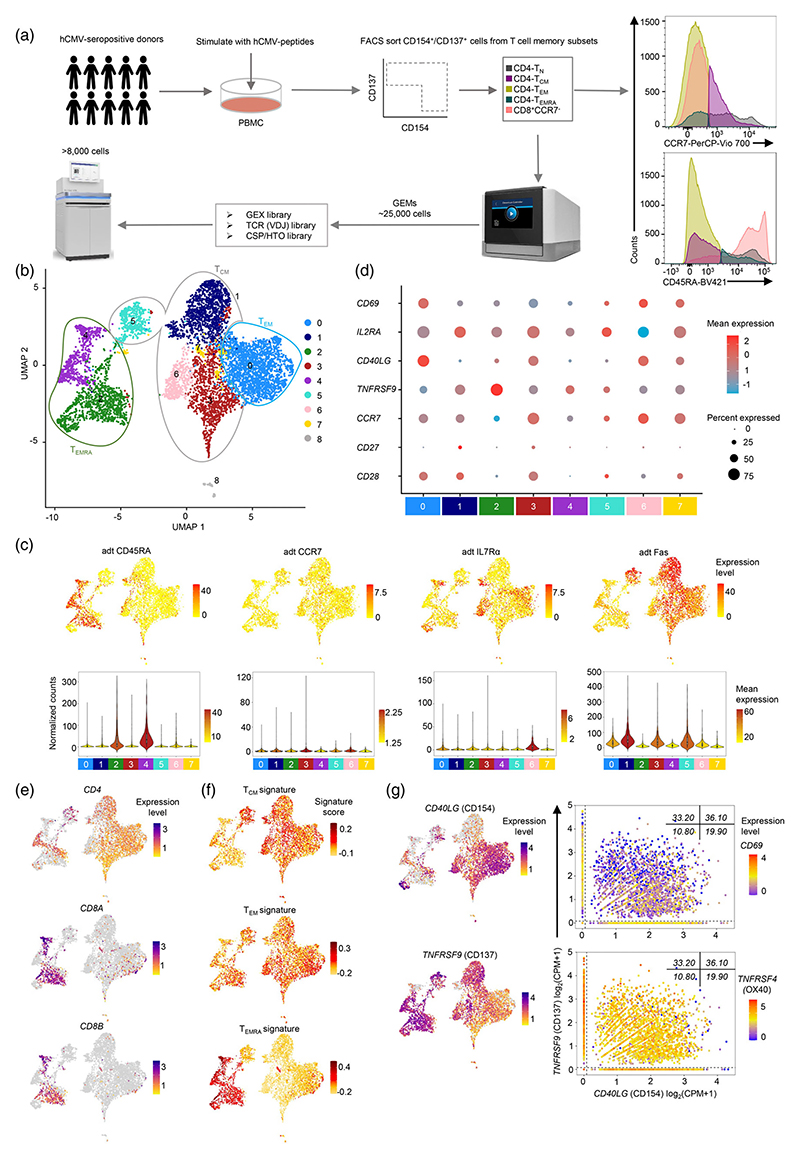
Single-cell transcriptomic analysis of hCMV-reactive memory T cell subsets. (a) Schematic showing the study overview for isolating and single-cell RNA-seq of hCMV-specific T cells using 10X Genomics platform. Histograms (on the right) show cells isolated using FACS for single-cell RNA-Seq from indicated T cell memory compartment from 10 donors. (b) 2D UMAP of single-cell RNA-Seq data from 6899 cells, using 2000 most variable transcripts, shows nine distinct clusters of cells of hCMV-specific memory T cells. (c) 2D UMAP plots (top) and violin plots (bottom) show the expression of the indicated protein (oligo-tagged antibodies; adt). Colour scale shows expression levels of individual cells (UMAP) or mean expression in each cluster (violin plot). Only cells (6067) obtained from Experiment 2 with 10 donors are shown. (d) Dot plots show the mean expression (colour) and percentage of expressing cells (size) for selected marker gene transcripts in each cluster. (e) 2D UMAP plots of single-cell RNA-Seq data show the expression of the *CD4, CD8A* and *CD8B* transcripts. Scale represents the expression level of the given transcript. Cells with zero counts for the indicated transcript are shown in grey colour. (f) 2D UMAP plots show the T_CM_, T_EM_ and T_EMRA_ signature score for each cell. (g) 2D UMAP plots (left) of single-cell RNA-Seq data show the expression of the *CD154* (top) and *CD137* (bottom) transcripts. Scale represents the expression level of the given transcript. Cells with zero counts for the indicated transcripts are shown in grey colour. Scatter plots (right) show the co-expression matrix for *CD154* (on *x*-axis), and *CD137* (on *y*-axis) with *CD69* or *TNFRSF4* (colour scale: mean expression) transcripts. For violin plots in (c) and dot plots in (d) cluster with less than 1% of the total cells is not shown (cluster 8 = 56 cells). All expression counts for transcripts are log_2_ normalized (CPM + 1). hCMV, human cytomegalovirus.

To identify and understand the heterogeneity and clonal diversity of the spectrum of hCMV-specific T cells across different memory subsets in an unbiased way, we performed single-cell transcriptomic (scRNA-Seq) and single-cell TCR repertoire analysis (scTCR-Seq) of >8000 hCMV-specific memory T cells (Figure 1a,b). To isolate the spectrum of hCMV-specific T cells independent of their cytokine profile and HLA restriction, we utilized the widely used ARTE assay, where the surface expression of co-stimulatory markers such as CD154 encoded by *CD40LG* and CD137 encoded by *TNFRSF9* is analyzed upon the engagement of TCR with cognate pMHC (peptide Major Histocompatibility Complex) complex [1, 3, 7, 40, 41, 46]. For single-cell omics experiments, we isolated T cells using fluorescence-assisted cell sorting (FACS) based on the surface expression of CD154 and CD137 upon stimulation of PBMCs from 10 hCMV-seropositive and high peptide responder individuals with peptide pools derived from immunodominant pp65 and IE-1 (Figures 1a and S1a–c). To compare the hCMV-specific memory T cells across known memory subsets, the peptide-responding T cells, from different CD4^+^ memory T cell compartments (naïve T cells [T_N_; CD45RA^+^CCR7^+^], central memory T cells [T_CM_; CD45RA^−^CCR7^+^], effector memory T cells [T_EM_; CD45RA^−^CCR7^−^], and effector memory T cells expressing CD45RA [T_EMRA_; CD45RA^+^CCR7^−^] [15]), were FACS sorted separately and pooled before performing single-cell omics experiments (Figures 1a and S1d). Further, considering the enrichment of CD4-CTLs (cytotoxic T lymphocytes) in the CD4-T_EMRA_ subset, to parallelly compare the hCMV-specific CD4-CTLs and CD8-CTLs we also included hCMV-reactive T cells from CD8-CTL compartments (CD8^+^CCR7^−^ [CD8-T_EM_ and -T_EMRA_]) in the single-cell omics experiments (Figures 1a and S1c,d) [4]. An unbiased clustering of 6899 hCMV-specific memory T cells (out of 8062 cells) that passed our quality control parameters (QC; please see methods section), identified nine distinct clusters based on transcriptomes (Figure 1b), and the clustering was not influenced by donors or the experimental batch (Figure S1e,f; Table S1). Different developmental T cell memory subsets were distinguished based on the surface expression of oligo-tagged antibodies (antibody-derived tags; adt) against CD45RA, CCR7, CD95 and IL7Rα as well as *CCR7, CD27, CD28* transcripts (Figure 1c,d; Table S2) [4, 15]. Transcripts for *CD4* were distributed across all clusters, while *CD8B* transcripts were majorly found in clusters 2 and 4 (Figure 1e). Cells in clusters 2 and 4 were mostly T_EMRA_ cells of both CD4 and CD8 origin as they expressed CD45RA (adt CD45RA) and lacked the expression of CCR7 protein (adt CCR7), as well as transcripts for *CCR7, CD28* and *CD27* (Figure 1c,d) [4, 15]. T_N_ cells were very few and were mostly found in cluster 6 (CD45RA^+^CCR7^+^IL7Rα^+^) (Figure 1c,d). T_CM_ and T_EM_ cells were distributed across multiple clusters (clusters 0, 1, 3, 5, 6, 7), with higher proportion of T_EM_ cells observed in cluster 0 (Figure 1c,d). These observations were independently confirmed by the enrichment of subset-specific gene signatures from T_CM_, T_EM_ and T_EMRA_, as well as common gene signatures from T_CM_-T_EM_ and T_EM_-T_EMRA_ [4] (Figures 1f and S2a; Table S3). Both CD4-T_EMRA_ and CD8-T_EMRA_ cells (clusters 2 and 4) majorly showed higher expression of *CD137* transcript, while other T_CM_ and T_EM_ clusters (0, 3 and 6) predominantly showed higher expression of *CD154* transcript, which was consistent with the protein expression pattern observed in flow cytometry analysis (Figures 1g and S1b,d). Interestingly many of the T cell activation markers showed dynamic expression amongst the clusters; *CD154* and *CD137* showed opposing expression and similar observations were made for *CD69* and *IL2RA* (encoding CD25) (Figure 1d,g). Further, co-expression analysis of various T cell activation markers in combination, confirmed the variability in expression (Figure 1g). Most strikingly, the *CD137*^hi^ *CD154*^lo^ cells showed low *CD69* expression, while the *CD154* and *CD137* co-expressing cells expressed similar amounts of *TNFRSF4* transcripts (encoding OX40) (Figure 1g). Taken together, these results show that our data well represents various memory subsets that show differential expression of T cell activation markers.

### hCMV-specific memory T cells are heterogeneous and can be classified into multiple functional T cell subsets

**Figure 2:**
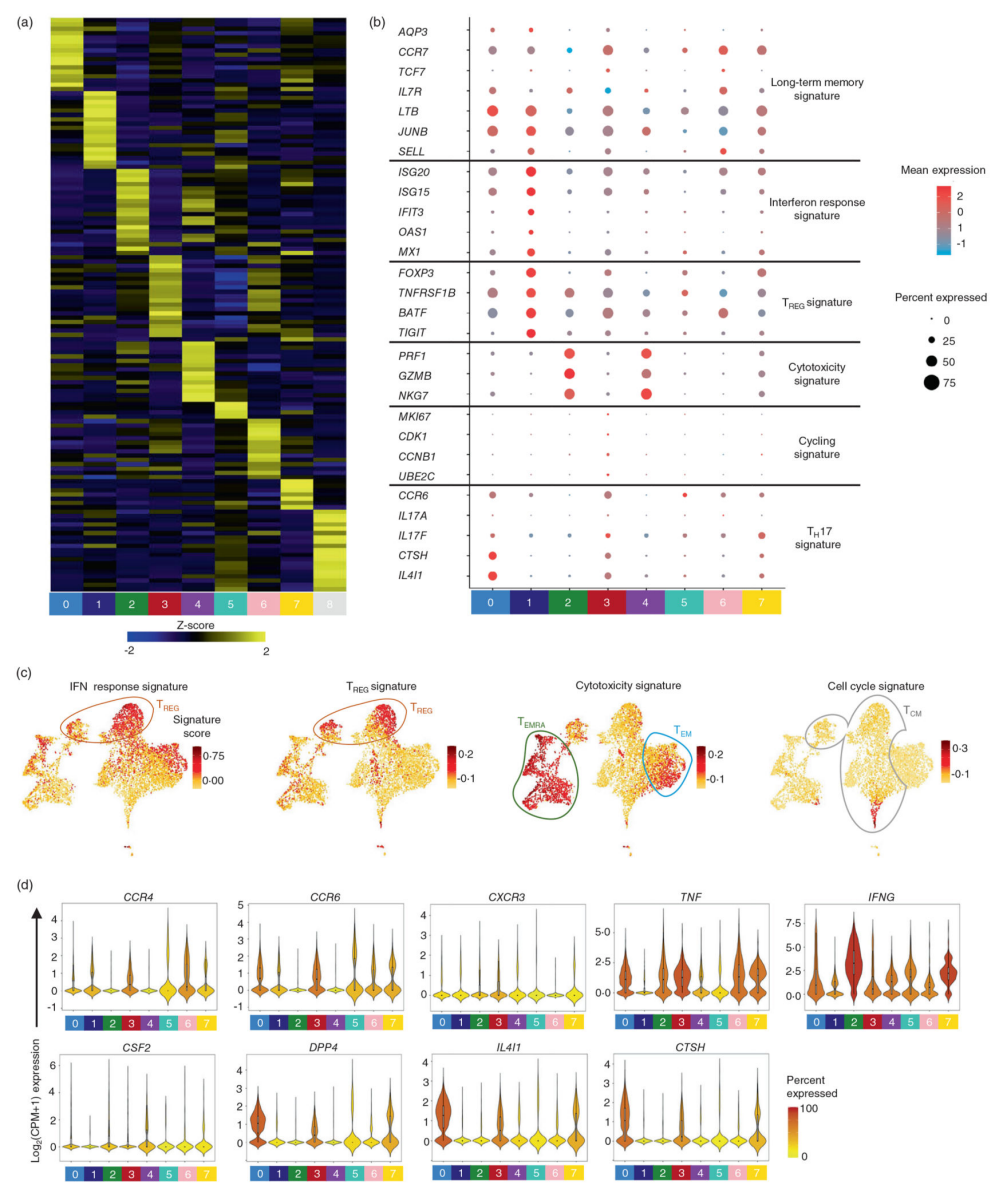
Single-cell transcriptomic analysis of hCMV-reactive T cell subtypes. (a) Heatmap shows the expression of the most significantly enriched transcripts in each cluster. The top 200 transcripts are shown based on adjusted *p* value <0.05, log_2_ fold change >0.25 and >10% difference in the percentage of cells expressing selected transcript between two groups of cells compared. Each column represents the average expression of all cells for a given cluster. Colour scale represents *z*-score for the expression of each transcript across clusters. (b) Dot plots show the mean expression (colour) and percentage of expressing cells (size) for selected marker gene transcript in each cluster. (c) 2D UMAP plots show the IFN response, T_REG_, cytotoxicity and cell cycle signature score for each cell. (d) Violin plots show the normalized expression level (log_2_(CPM + 1)) of the indicated differentially expressed transcript. Colour scale shows the percentage of cells expressing the indicated transcript for each cluster. For (b and d), clusters with <1% of the total cells are not shown (cluster 8 = 56 cells). hCMV, human cytomegalovirus; IFN, interferon.

The differential expression analysis of transcripts amongst the nine clusters of hCMV-specific T cells, identified cluster-specific expression of transcripts, revealing many functional T cell subsets (Figure 2a,b; Table S4). Multiple clusters showed enrichment for long-term memory markers such as *AQP3, CCR7, TCF7, IL7R, LTB, JUNB*, and *SELL* (Figure 2a,b) [3, 4]. Cluster 1 showed enrichment for *FOXP3, BATF, TIGIT* and *TNFRSF1B* transcripts that are known to be expressed in T regulatory cells (T_REG_) (T_REG_ cluster) (Figure 2b). Interestingly, cluster 1 (T_REG_ cluster) also showed enrichment of transcripts encoding interferon (IFN) response signature genes (*ISG20, ISG15, IFIT3, OAS1* and *MX1*) (Figure 2b). In an independent analysis, cluster 1 cells showed a higher enrichment signature score for overall T_REG_ and IFN response signature gene sets (Figure 2c; Table S3), suggesting that cells in cluster 1 are T_REG_ cells and are possibly responding to IFN signaling (T_REG-IFN_). Actively cycling cells were found in cluster 3, one of the clusters enriched for T_CM_ signatures (T_CM_-cluster) (Figure 2b,c; Table S3) [47]. Cells in clusters 2 and 4, enriched for T_EMRA_-specific gene sets (T_EMRA_-clusters), expressed transcripts encoding cytolytic molecules such as *GZMB, PRF1* and *NKG7* and showed enrichment for cytotoxicity gene signatures (Figures 1f, 2b,c and S2a; Tables S3 and S4) [4], suggesting that clusters 2 and 4 are cytotoxic T cell clusters (T_CTL_-clusters). The presence of a mixture of cells expressing *CD4* and *CD8* transcripts in both the cytotoxic clusters 2 and 4 (T_CTL_-clusters) suggests possible similarities between CD4-CTLs and CD8-CTLs (Figure 1e).

We next interrogated the single-cell transcriptome of hCMV-specific T cells for functional T helper (T_H_) cell subtypes such as T_H_1, T_H_17 and T_H_1/17, which are known to have a role in viral infections [3, 48]. Multiple clusters showed expression of T_H_ subset-specific transcripts: T_H_17 (*CCR4, CCR6, IL4I1* and *CTSH*), T_H_1 (*CXCR3, TNF, IFNG* and *CSF2*) and T_H_1/17 (*CXCR3* and *DPP4*) [9, 10] (Figure 2d; Table S4). Interestingly, we found several clusters co-expressing transcripts from different T_H_ subsets, suggesting each cluster may have cells belonging to more than one T_H_ subtype. Independently, module enrichment analysis using T_H_ subset-specific gene sets corroborated these results (Figure S2b; Table S3). The T_H_1-specific gene sets were enriched in clusters 2 and 4, the cytotoxic clusters (T_CTL_-clusters) (Figure S2b). Considering there are several overlapping genes between T_H_1 and cytotoxic gene sets, this observation is in alignment with the known literature [4, 9, 10]. T_H_17 signature genes showed the highest enrichment in cluster 0, followed by clusters 7, 3 and 1. T_H_1/17 signatures were mostly enriched in cluster 0, followed by clusters 5 and 7 (T_CM_ and T_EM_ clusters) (Figure S2b) [9, 10]. Taken together, these observations indicate that each hCMV-specific T_H_ subset is at different developmental stages of memory (long-term memory and effector memory) and clustering was predominantly influenced by the developmental stages of memory T cells.

### TCR clonally expanded hCMV-specific T cells are majorly observed in cytotoxic clusters

**Figure 3:**
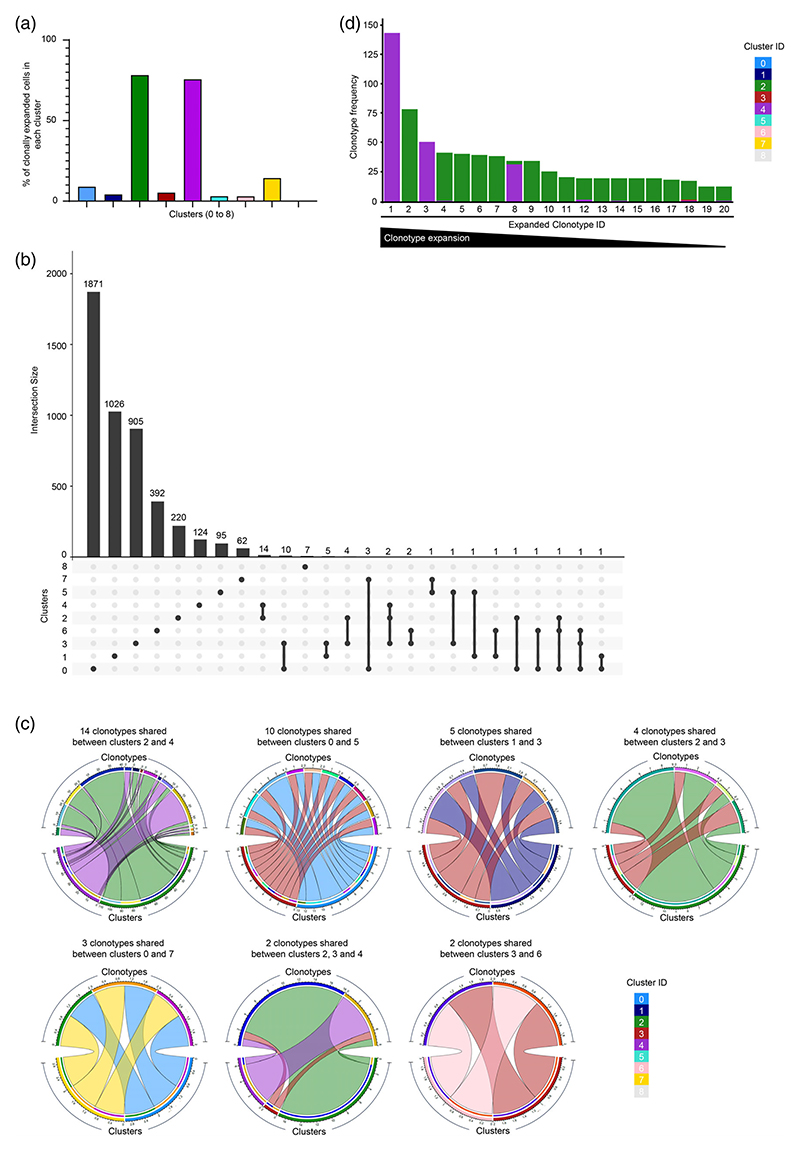
TCR repertoire analysis of CMV-specific memory T cells. (a) Bar graph shows the percentage of cells with expanded clonotypes (frequency ≥2) across nine clusters. (b) UpSet plot shows the TCR clonotypes (frequency on *y*-axis) and sharing of the clonotypes (bottom, connected dots) across the nine clusters. (c) Circos plots show the sharing of the indicated clonotypes between different clusters. (d) Bar graph shows the frequency of 20 most expanded clonotypes across different clusters. CMV, cytomegalovirus; TCR, T cell antigen receptor.

To understand the TCR clonal diversity amongst the hCMV-specific T cells, we analyzed the single-cell TCR-Seq data generated from the same cells. We recovered TCRs from 83.56% (5765 out of 6899 cells) of the total QC-ed cells in our data set, of which 21.40% (1234 out of 5765 cells) were clonally expanded (Figure 3a; Table S5). Several clusters shared TCR clonotypes, indicating a shared developmental lineage between these clusters (Figure 3b). A maximum of 14 clonotypes were shared between clusters 2 and 4 (T_CTL_-clusters), followed by clusters 0 and 3 (T_EM_ and T_CM_ clusters) sharing 10 clono-types (Figure 3b,c). Cells in clusters 2 and 4 (T_CTL_-clusters) showed the highest clonal expansion in each individual donor as well as when analyzed across all the donors with overall ~78% and ~ 76% of the cells being expanded, respectively (Figures 3a and S3a; Table S5). As high as 76% of the total expanded cells (938 of 1234) were found in cluster 2 (50%) and cluster 4 (~26%) (T_CTL_-clusters) (Table S5). A few top expanded clonotypes were found in as many as 144, 79 and 51 cells from clusters 2 and 4 (T_CTL_-clusters), indicating the preferential expansion of a few specific clonotypes (Figures 3d and S3b). Interestingly, close to 58% of the expanded cells carried only 20 clonotypes (Figure 3d). Both CD4-CTLs and CD8-CTLs (T_EMRA_) are shown to be clonally expanded in several diseases including CMV in both humans and animal models [2–4, 49, 50]. Clonal expansion and enrichment for cytotoxic signature in T_CTL_-clusters (clusters 2 and 4) emphasize the role for cytotoxic cells in hCMV-specific protective immune response [16–20].

### Single-cell transcriptome analysis of hCMV-specific T_REG_ cells

**Figure 4:**
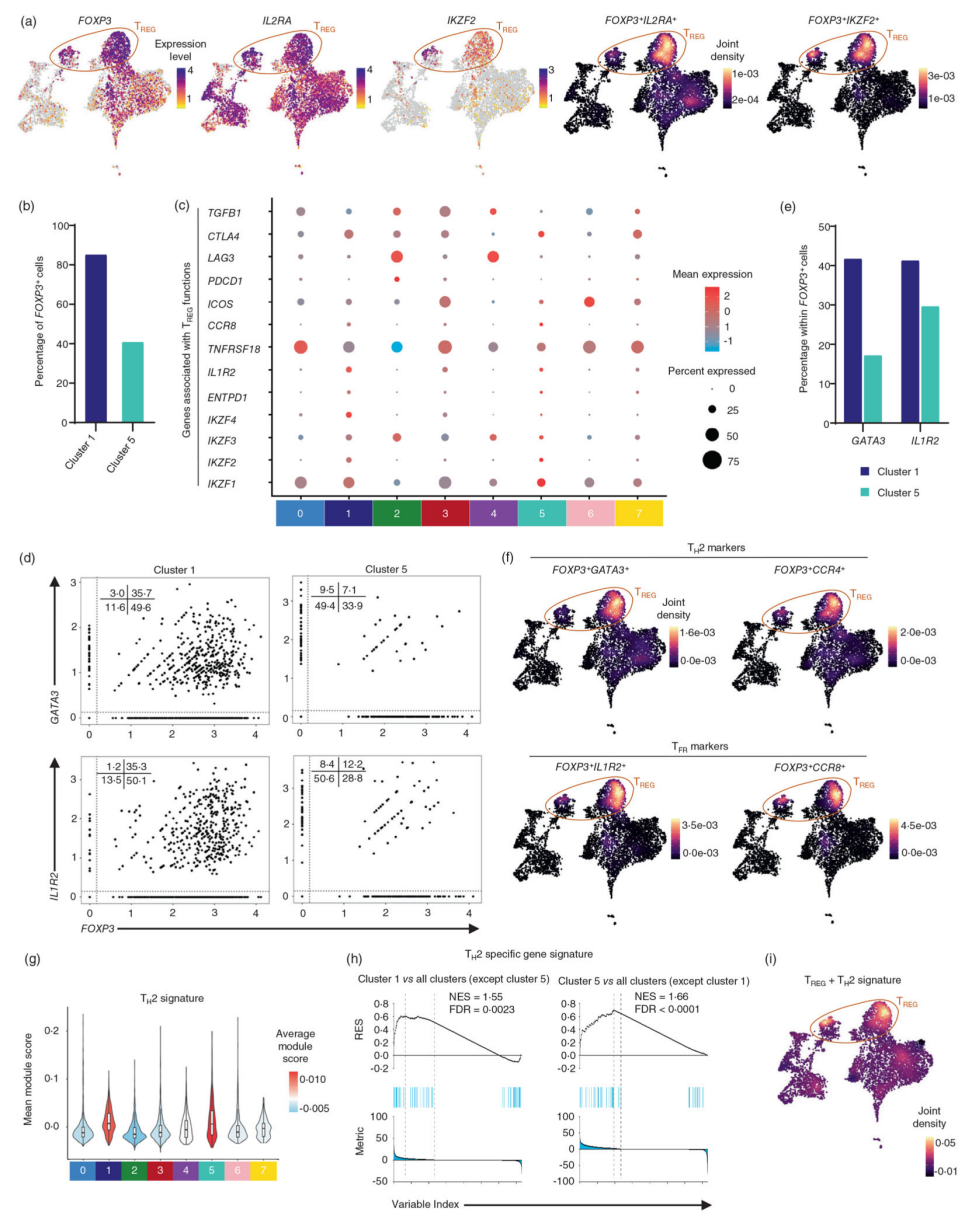
Single-cell transcriptomic analysis of hCMV-reactive T_REGs_. (a) 2D UMAP plots of single-cell RNA-Seq data show the expression of the *FOXP3, IL2RA, IKZF2* (left) and joint expression of *FOXP3* with *IL2RA* and *FOXP3* with *IKZF2* (right two plots) transcripts. Scale represents log_2_ normalized (CPM + 1) counts for expression values and scaled joint density for joint expression plots. Cells with zero counts for the indicated transcripts are shown in grey colour. (b) Bar graph shows the percentage of cells expressing *FOXP3* transcript (log_2_(CPM + 1) > 0) in clusters 1 and 5. (c) Dot plots show the mean expression (colour) and percentage of expressing cells (size) for selected marker gene transcripts in indicated clusters. Clusters with <1% of the total cells are not shown (cluster 8 = 56 cells). (d) Scatter plots showing normalized co-expression level (log_2_(CPM + 1)) between *FOXP3* and *GATA3* and *FOXP3* and *IL2RA* transcripts in the indicated clusters. (e) Bar graph shows the percentage of cells expressing *GATA3* or *IL1R2* transcripts (log_2_(CPM + 1) > 0) within *FOXP3* transcript expressing cells in clusters 1 and 5. (f) 2D UMAP plots show joint expression of *FOXP3* + *GATA3, FOXP3* + *CCR4* and *FOXP3* + *IL1R2, FOXP3* + *CCR8*. Scale represents scaled joint density. (g) Violin plot shows the module enrichment score for T_H_2-specific gene set across eight clusters. Colour scale indicates the mean module score. Clusters with <1% of the total cells are not shown (cluster 8 = 56 cells). (h) Gene set enrichment plot for T_H_2-specific gene sets in clusters 1 and 5 versus all the other clusters. (i) 2D UMAP plot shows joint expression of T_H_2-specific gene set and allergy-induced T_REG_-specific gene set. Scale represents joint density. FDR, false discovery rate; hCMV, human cytomegalovirus; NES, normalized enrichment score; RES, running enrichment score.

T_REGs_ are characterized by the higher expression of CD25 (IL2RA) and the transcription factor FOXP3, but a lower expression of IL7Rα (CD127) in humans [51]. CMV-specific T_REGs_ have been reported in both mouse models and humans following primary infections and are shown to produce IL10, a suppressive cytokine [2, 6, 36–39, 52–54]. However, the detailed molecular signatures of hCMV-specific memory T_REGs_ have not been well described. The transcript encoding the T_REG_-specific transcription factor FOXP3 was one of the DE transcripts in clusters 1 (85% of cells) and 7 (Figures 2b and 4a,b; Table S4), however though not a DE transcript, a portion of cells from cluster 5 showed higher *FOXP3* expression (41% of cells), while various other clusters (clusters 0 and 3; T_EM_ and T_CM_ clusters) showed lower levels of *FOXP3* expression (Figure 4a,b). Considering FOXP3 can be transiently expressed on TCR-activated cells, we examined the co-expression of other T_REG_-related genes and *FOXP3*. The cells co-expressing *FOXP3*-*IL2RA*, and *FOXP3*-*IKZF2* were enriched in cluster 1 and portion of cluster 5, and as expected they showed lower expression of IL7Rα (Figures 1c and 4a) [51]. Clusters 1 and 5 were also characterized by the higher expression of other T_REG_-specific transcripts such as *TIGIT, BATF, TNFRSF1B* and the Ikaros Zinc Finger (IkZF) transcription factor (TF) family members *IKZF1* encoding Ikaros, *IKZF2* encoding Helios, *IKZF3* encoding Aiolos, *IKZF4* encoding Eos (Figures 2b and 4c). The overall T_REG_ gene signature was enriched in cluster 1 and fraction of cluster 5 (T_REG_ clusters) (Figure 2c). Taken together, these results show that T_REG_ cells are predominantly in cluster 1 and portion of cluster 5 and the cells from clusters 0 and 3 are potentially non-T_REG_ cells, transiently expressing low levels of *FOXP3* in response to TCR activation. We next interrogated if the hCMV antigen-specific T_REGs_ (cluster 1 and portion of cluster 5) in our data set were indeed memory T_REGs_. Based on the expression of CD45RA, the FOXP3^+^ T_REGs_ have been classified as resting or naïve T_REGs_ (FOXP3^lo^CD45RA^+^) and effector T_REGs_ (FOXP3^hi^CD45RA^−^) [55, 56]. Further, the effector T_REGs_ have been classified as central and effector memory cells based on the expression of CCR7, CD27 and CD28 [57]. The T_REGs_ in our data set were characterized by the lack of expression of CD45RA, and a moderate expression of CCR7, *CD27* and *CD28*, indicating that they are mostly central and effector memory T_REGs_ rather than naïve T_REGs_ (Figure 1c,d).

Although T_REGs_ expressing FOXP3, are known for their suppressive function, many reports have shown the presence of T cells expressing FOXP3 transiently, in response to TCR activation that are non-suppressive in nature. Hence, we assessed if the hCMV-specific T_REGs_ in our data set are expressing markers associated with the suppressive nature of T_REGs_. The molecular signatures of T_REGs_ found in clusters 1 and 5 indicated their potential suppressive nature, compared to cells expressing low levels of *FOXP3* observed in clusters 0 and 3 (T_EM_ and T_CM_ clusters) (Figure 4a,c). The T_REGs_ in clusters 1 and 5 were *CD137*^+^ *CD154*^*lo*^, the activation-induced characteristic of T_REGs_ implicative of epigenetically stable antigen-activated T_REGs_ that retain the high suppressive potential in humans after in vitro or ex vivo activation (Figure 1d,g) [58]. The T_REGs_ in clusters 1 and 5, showed higher expression of *TIGIT, TNFRSF18* (GITR) and *CTLA-4*, genes that are associated with T_REGs_ containing high-affinity TCRs, further suggesting their antigen-specificity (Figures 2b and 4c). The T_REGs_ in clusters 1 and 5 also showed higher expression of *ENTPD1* (CD39), an ectoenzyme ATP apyrase, *CCR4, CTLA4*, Helios (*IKZF2*) and FAS (CD95), molecules that are reported to be associated with effector and suppressive function (Figures 1c, 2d and 4c) [59–61]. A suppressive function of Helios in T_REGs_ has been suggested, where Helios^+^FOXP3^+^ T_REG_ cells produced lower amounts of T_H_17-specific cytokine, IL17, compared to Helios^−^FOXP3^+^ T_REG_ cells, hence promoting the formation of T_REGs_ rather than T_H_17 subtype [61]. Consistent with these observations, we found that clusters 0 and 3 that expressed *IL17F* and *IL17A*, expressed low levels of *FOXP3*, but lacked the expression of *IKZF2* (Helios) and these cells showed higher expression of *CD154* and lower expression of *CD137* (Figures 2b and 4a,c) [58, 61]. Taken together, these observations suggest that the low *FOXP3*-expressing-cells in clusters 0 and 3 are potentially the induced/transient T_REGs_ that are possibly expressing *FOXP3* transiently in response to activation and are non-suppressive in nature. Conversely, T_REG_ cells in clusters 1 and 5 are *Helios*^+^*FOXP3*^+^, and did not express *IL17A* and *IL17F*, indicative of their suppressive nature (Figures 2b and 4a,c). The lack of expression of exhaustion markers *PDCD1* and *LAG3* further suggests these are not associated with anergy or exhaustion (Figure 4c). Taken together, these results provide multiple evidences to suggest that the T_REGs_ in clusters 1 and 5 are effector memory T_REGs_ and are potentially suppressive in nature. One of the mechanisms through which T_REGs_ execute their suppressive function on effector T cells is through cytokines such as IL10, TGFβ and IL35 (reviewed in reference [62]). The T_REGs_ in cluster 1 lacked or showed low expression of these suppressor cytokine transcripts *TGFB1, IL10* and *IL35*, indicating that cells in cluster 1 may not be actively suppressing the hCMV-specific effector T cells, at least in the experimental setting used in the study (Figure 4c; Table S4).

We next interrogated if the T_REG_ subsets in our data set, that showed enrichment for molecules linked to their suppressive function, are not suppressive towards hCMV-specific effector T cells, then which cell types are they targeting in hCMV-specific immunity? Studies using mouse models have shown that T_REGs_ can show remarkable functional plasticity and execute their suppressive function by expressing the TF and the chemokine receptor of the target T_H_ subtypes [14, 63–66]. For example, the T_REGs_ suppressive towards T_H_1 will express T-bet (encoded by *TBX21*) and CXCR3 (T_H_1-T_REGs_), while the ones towards T_H_2 will express GATA3 and CCR4 (T_H_2-T_REGs_), albeit at a reduced level compared to the T_H_ subtype itself, without necessarily themselves making the T_H_ subtype-specific cytokines [14, 64–66]. Interestingly, the differential gene expression analysis revealed that the T_REGs_ in clusters 1 and 5 are characterized by the higher expression of T_H_2-specific TF *GATA3* and chemokine receptor *CCR4* along with *FOXP3* (Figure 4d–f). GATA3 has been shown to be important for FOXP3 expression and T_REG_ functions [65]. Hence, we independently examined the overall T_H_2 subset-specific gene signatures using module enrichment score and GSEA. We found that both clusters 1 and 5 are enriched for T_H_2 subset-specific gene signatures (Figure 4g,h), suggesting the T_REGs_ in clusters 1 and 5 are most probably T_H_2-like T_REGs_. A recent study that analyzed the T cells in pan cancers has also shown the presence of T_H_2-like T_REGs_ that were marked by a higher expression of *GATA3* [67]. Clusters 1 and 5 were also characterized by the enrichment of IFN response signature genes (T_REG-IFN_) (Figure 2b,c). In asthma and house dust mite allergy, both T_H_ and T_REG_ subsets enriched for IFN response gene sets showed dampened T_H_2 response in healthy individuals with no prior history of allergy or asthma [5]. Interestingly, in our data set, a joint enrichment analysis using the house dust mite allergy-specific T_REG_ gene signature and T_H_2-specific gene signature showed enrichment in clusters 1 and 5 (Figure 4i) [5]. Taken together, these results suggest that T_REGs_ in clusters 1 and 5 are likely T_H_2-T_REGs_ with IFN response signatures that are potentially suppressive towards T_H_2. The T follicular regulatory cells (T_FRs_) suppress T_FH_ responses and are characterized by the expression of IL1R2 and CCR8 [3, 68, 69]. The hCMV-reactive T_REG_ cells in clusters 1 and 5 co-expressed *IL1R2* and *CCR8* transcripts along with *FOXP3* transcripts, hence, may represent T_FR_ cells (Figure 4d–f) [3, 68, 69]. These interesting observations warrant further investigation to understand the relevance of T_H_2-T_REGs_ and T_FRs_ in CMV immunity, especially when hCMV-reactive conventional (non-T_REGs_) T_H_2 and T_FH_ cells were absent in our data set.

### hCMV-reactive CD4-CTLs and CD8-CTLs show similar transcriptomic profile

**Figure 5:**
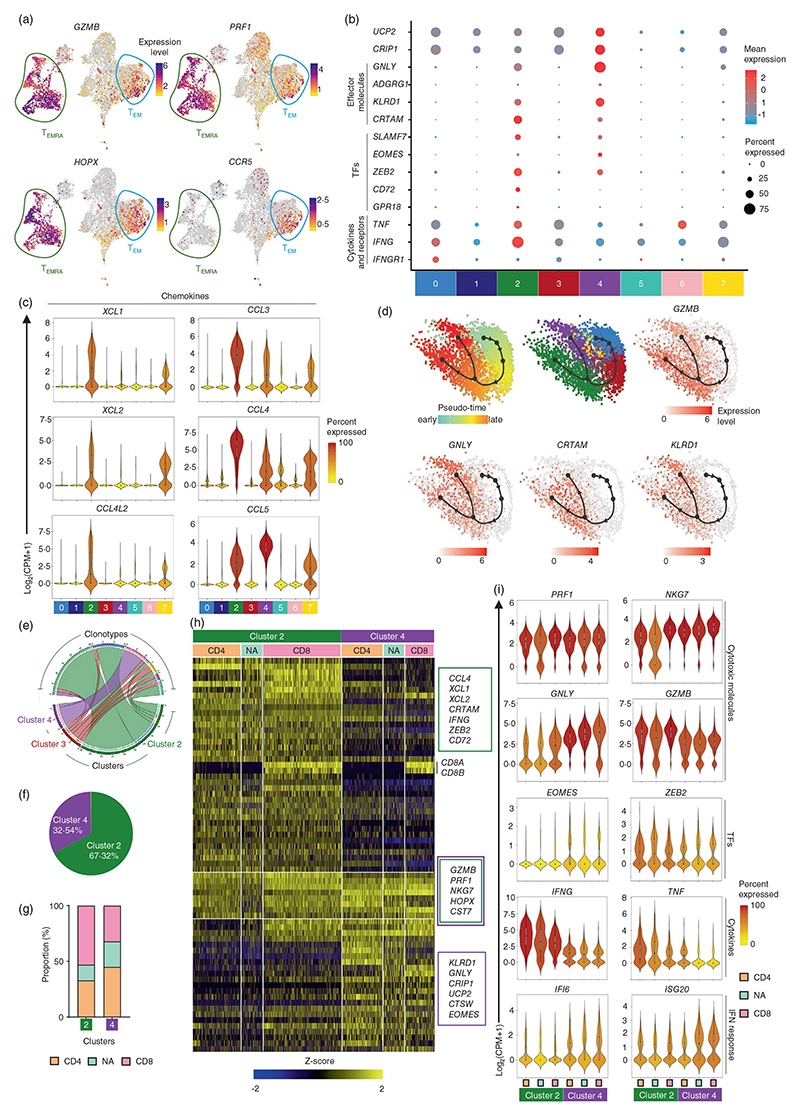
Single-cell transcriptomic analysis of hCMV-reactive CD4-CTLs and CD8-CTLs. (a) 2D UMAP plots of single-cell RNA-Seq data show the expression of the indicated transcripts. All expression counts for transcripts are log_2_ normalized (CPM + 1). Cells with zero counts for the indicated transcripts are shown in grey colour. (b) Dot plots show the mean expression (colour) and percentage of expressing cells (size) for selected marker gene transcripts in each cluster. (c) Violin plots show the normalized expression level (log_2_(CPM + 1)) of the indicated differentially expressed transcripts across eight clusters. Colour scale shows the percentage of cells expressing the indicated transcript for a given cluster. (d) Trajectory analysis using slingshot shows the distribution of cells from indicated clusters (middle) on a pseudo-scale (left), and expression levels (scale) of the *GZMB, GNLY, CRTAM* and *KLRD1* transcripts. (e) Circos plot shows the clonotypes from cluster 3, which are shared with clusters 2 and 4. (f) Pie chart shows the distribution of percentage of cells expressing the top 20 expanded clonotypes in clusters 2 and 4. (g) Stacked bar graph shows the proportion of CD4, CD8 and NA (not assigned) T cells in each indicated cluster (refer to Materials and Methods section for classification of cells). (h) Heatmap shows the normalized expression of 50 differentially expressed transcripts from each, clusters 2 and 4 when compared to the rest of the clusters. (i) Violin plots show the normalized expression level (log_2_(CPM + 1)) of the indicated differentially expressed transcript across indicated T cell subsets in clusters 2 and 4. Colour scale shows the percentage of cells expressing the indicated transcript. For (b and c), clusters with <1% of the total cells are not shown (cluster 8 = 56 cells). hCMV, human cytomegalovirus.

Majority of the cells in clusters 2 and 4 and fraction of cells in clusters 0 and 7 were characterized by the high expression of transcripts encoding cytolytic molecules such as Granzyme B (*GZMB*), Perforin (*PRF1*), Granulysin (*GNLY*), and transcription factors *HOPX* and *ZEB2* and showed enrichment for cytotoxicity and T_EMRA_-specific signature genes (Figures 1f, 2b,c and 5a,b) [3, 4, 16–20]. Interestingly, these cytotoxic clusters could not be differentiated based on the expression of *CD4* or *CD8A* and *CD8B* transcripts, but by other cytotoxicity-related molecules (Figures 1e and 5a–c). The cytolytic granules have been shown to contain chemokines such as CCL3, CCL4 and CCL5 in addition to the classical mediators of cytotoxicity (GZMB, PRF1 and GNLY), in both viral and bacterial infections, indicating their role in cytolysis [70, 71]. Cells in cluster 2 showed higher expression of the transcripts encoding the cytokines such as *TNF* and *IFNG*, chemokines such as *CCL3, CCL4, CCL4L2, XCL1* and *XCL2*, while cluster 4 showed higher expression of *CCL5* (Figure 5b,c). The chemokine receptor *CCR5*, the receptor for chemokines *CCL3, CCL4* and *CCL5*, and *IFNGR1*, encoding the receptor for IFNG, was expressed by the cytotoxic cells in cluster 0, suggesting these cells may be responding to the cytokines and chemokines made by the cells in cytotoxic clusters 2 and 4 (T_CTL_-clusters) and are potentially the intermediate population (Figure 5a,b; Table S4) [72]. This is further supported by the single-cell trajectory analysis, where on the pseudo time scale, cluster 0 appears before clusters 2 and 4 (Figure 5d). These results suggest that the cytotoxic cells in cluster 0 are potentially an intermediate population in the CTL lineage and these cells are mostly T_EM_ cells as they mostly lack the expression of CD45RA and CCR7 (Figure 1c).

Compared to cluster 4, cluster 2 cells showed higher expression of *SLAMF7, ZEB2, GZMB, GPR18, CD72, PDCD1* (PD1) and *CRTAM* that are known to be expressed in terminally differentiated effector cells in viral infections and various cancers [3, 73–76] (Figures 2b, 4c and 5b,d). On the contrary, cluster 4 was characterized by the higher expression of *GNLY, EOMES, ADGRG1* (GPR56), *KLRD1, UCP2* and *CRIP1* (Figures 2b and 5b,d), which are mostly not associated with terminally differentiated effector T cells. For example, CRIP1 has been shown to be anti-apoptotic in cancers and the antigen-specific cells expressing UCP2 survive better in HIV infection through metabolic reprogramming [77, 78], suggesting the possible role of these genes in providing survival advantage to cells in cluster 4. A coordinated expression of CCL5 followed by GNLY and PRF1 provided host-defence against *Mycobacterium tuberculosis* (Mtb) infection, where the expression of CCL5 attracted the Mtb-infected macrophages that in-turn triggered the expression of GNLY and PRF1 in CD8 T cells [79]. Further, a delayed onset of cells expressing GNLY has been shown in multiple infections [80, 81]. These results suggest that the cytotoxic cells could be at different stages of their development or effector function. The differential expression of many of these cytolytic molecules between clusters 2 and 4 implies that the cells in cluster 2 are potentially the terminal effector cells while those in cluster 4 are pre-effector cells. This observation is further strengthened by the TCR analysis (Figures 3b–d, 5e,f and S3b). The 20 most expanded clonotypes were found majorly in clusters 2 and 4 (Figures 3d and S3b). Cluster 2 is relatively more clonally expanded than cluster 4, where over 67% of the cells harbouring the 20 most expanded clonotypes were found in cluster 2 (Figure 5f). The trajectory analysis also showed that on a pseudo-time scale, clusters 2 and 4 appear to bifurcate from a common origin of cluster 0 (Figure 5d). Further, the pathway enrichment analysis of genes DE between clusters 2 and 4 revealed an enrichment for cytokine and chemokine response pathways in cluster 4, while cluster 2 showed enrichment of pathways associated with pathogen-induced cytokine storm (Figure S4a,b). The effector T cells utilize glycolysis, as the preferred metabolic pathway that plays an important role for cytokine secretion and lytic nature of these effector cells [82–84]. The cells in cluster 2 showed enrichment for many metabolic pathways associated with effector cells such as glycolysis and HIF1α signalling as well as pathways related to anti-viral responses (Figure S4a). Taken together, these combined analyses of the transcriptome, TCR clonotype expansion and sharing along with the trajectory analysis provide the evidence to support the idea that cluster 4 cells are T_CTL_-pre-effectors while cluster 2 cells are T_CTL_-effectors.

Next, to assess if the observed differences between the CTL clusters 2 and 4 are influenced by the proportion and the characteristics of CD4-CTLs and CD8-CTLs, we compared the expression of these transcripts between CD4 and CD8 T cells within clusters 2 and 4. The proportions of CD4 and CD8 T cells within clusters 2 and 4 were comparable (Figure 5g), indicating that the disproportionate distribution of CD4^+^ and CD8^+^ T cells is not the cause of the observed differences between clusters 2 and 4. Overall, the transcriptome profile of CD4, CD8 or cells that could not be assigned as CD4 or CD8 T cells (NA) within each cluster did not vary except for *CD8A* and *CD8B* transcripts (Figure 5h,i). Transcripts such as *PRF1* and *NKG7* that showed comparable expression between clusters 2 and 4, followed the same pattern even when these two clusters were further sub-divided based on the CD4 and CD8 T cell sub-type (Figure 5h,i). The transcripts that showed higher expression in cluster 2 (*XCL1, ZEB2, CRTAM* and *GZMB*) and cluster 4 (*CCL5, GNLY* and *EOMES*), followed similar patterns of expression in CD4 and CD8 T cells within each of these effector and pre-effector clusters (Figure 5h,i). Both CD4 and CD8 T cells from cluster 4 showed higher expression of IFN response genes *ISG20* and *IFI6*, while those in cluster 2 showed higher expression of cytokines *TNF* and *IFNG* (Figure 5h,i). We observed that the cells in clusters 2 and 4 have distinct gene expression patterns that are not influenced by either CD4 or CD8 T cell proportion (Figure 5h,i). Overall, our data have revealed that both hCMV-reactive CD4-CTLs and CD8-CTLs share similar transcriptome profiles and can be classified as effectors and pre-effectors of CTLs.

## Discussion

A significant world population carries the latent hCMV infection, without showing any symptoms or causing the disease. However, latent infection poses a huge risk of reactivation in immune-compromised individuals such as those undergoing transplant or infants born to hCMV seropositive mothers. Although there is very little risk of the disease occurrence in immune-competent individuals, the latent CMV infection puts a huge burden on the immune system, especially for the elderly, where significant proportions of the T cells are found to be hCMV-specific [50]. Identifying and understanding the characteristics of the hCMV-specific T cell immune memory components in individuals carrying the virus in latent form, representing protective immune response, can be of high value to design therapy or vaccines to control CMV infection upon reactivation or maternal to fetal transfer.

Our understanding of ex vivo, antigen-specific human T cell responses to pathogens such as hCMV remains limited, due to the rarity of the cells, the complexity of the biology, and technical challenges of identifying overall antigen-specific CD4^+^ memory T cells, since they can be extremely heterogeneous in the expression of activation associated markers and cytokines. An added layer of complexity comes from HLA-restriction of individuals and the prior knowledge about the immune-dominant peptide, limiting the use of pMHC multimers. Most studies have focused on isolating the total CD4^+^ or CD8^+^ T cells reactive to hCMV, without considering the differences in memory proportions that can inadvertently bias the analysis towards analyzing the predominant memory compartment within CD4^+^ T cells (T_CM_ or T_EM_) and CD8^+^ T cells (T_EMRA_). Our strategy to isolate antigen-specific T cells based on the combination of TCR activation-dependent markers (CD154 and CD137) across different memory subsets followed by high-resolution single-cell RNA-Seq paired with single-cell TCR analysis allowed us to interrogate and compare a larger pool of hCMV (pp65 and IE-1)-reactive cells from different memory subsets across 10 seropositive and peptide-responsive donors. This unbiased approach enabled us to overlay the heterogeneity in T cell memory subsets classified based on developmental stages and functional subsets along with their TCR-dependent activation markers. The expression of CD154 and CD137 in different sets of cells that further differentially co-expressed one or more T cell activation-associated markers, such as CD69, CD25, OX40, warrants careful selection of activation markers while analyzing antigen-specific cells ex vivo. In this study, we observed that though activated (based on the expression of transcripts encoding CD154, CD137, CD69, CD25 and OX40), most cells, with the exception of effector cells, did not actively make cytokines and those that made also showed heterogeneity for the cytokine profile. Many studies previously have also relied on cytokine profiles to analyze ex vivo isolated antigen-specific cells and hence have mostly analyzed effector memory T cells.

The single-cell RNA-Seq of a large number of hCMV reactive cells isolated from 10 different donors, identified several interesting functional subsets: T_H_17, T_REGs_, CD4-CTLs and CD8-CTLs. Of particular interest were CD4- and CD8-CTLs, which clustered together. The two cytotoxic clusters observed could not be distinguished based on the CD4- and CD8-T cell-specific transcripts *CD4* or *CD8A*/*CD8B*, but on the basis of other cytotoxic molecules that were linked to different states of effector functions. The cluster that was predominantly making cytokine and chemokine (*IFNG, TNF, XCL1, XCL2, CCL3* and *CCL4*) also expressed transcription factor (TF) *ZEB2*, the TF known to be associated with late effector function [4, 76]. The cytotoxic cells in both clusters 4 and 0 appeared to respond to the cytokines and chemokines by expressing their receptors (*IFNGR1* and *CCR5*). However, the cytotoxic cells across these clusters expressed molecules linked to cytolytic granules (*PRF1, GZMB* and *GNLY*), and transcription factor *HOPX*. The differential expression of many of the molecules linked to cytotoxicity and effector functions, suggests the effector and pre-effector-like subsets. These subsets, possibly at different developmental states, might exist to ensure a continuity in immune response during the entire course of the infection.

Another interesting discovery in our data set is the identification of T_REGs_ with suppressive ability that showed enrichment for the IFN response gene signatures (T_REG-IFN_) which has been shown to be associated with a dampened T_H_2 response in allergy [5]. The enrichment of T_H_2 signatures as well as the expression of *GATA3* and *CCR4*, the T_H_2-associated transcription factor and chemokine receptor by the T_REGs_, further suggested that these could be suppressive towards the T_H_2 subset. Although a very recent study in pan-cancer has also identified this subset, to the best of our knowledge, presence of T_REGs_ enriched for IFN response genes has not been reported earlier in viral infections in humans [67]. Based on these observations, we hypothesize that during the initial stages of the infection, where the expansion of effector cells is necessary, T_REGs_ may not be executing their suppressive function towards these effectors, instead, suppressing other subtypes of no relevance to the disease. However, further kinetic studies in both animal models and longitudinal human cohorts of different types of diseases, such as viral and bacterial infections, allergies and cancers, are necessary to verify or refute this hypothesis.

## Supplementary Material

Supplementary figures

## Data Availability

Scripts will be made available on GitHub upon request. Sequencing data for this study is deposited onto the Gene Expression Omnibus with accession number GSE235604.
