# Supplementary figures and images for: Single-cell transcriptomic and T cell antigen receptor analysis of human cytomegalovirus (hCMV)-specific memory T cells reveals effectors and pre-effectors of CD8+- and CD4+-cytotoxic T cells
Source: Immunology. Author manuscript; Available in PMC 2024 Jul 1. (PMC7616077; doi:10.1111/imm.13783)

**FIGURE S1.**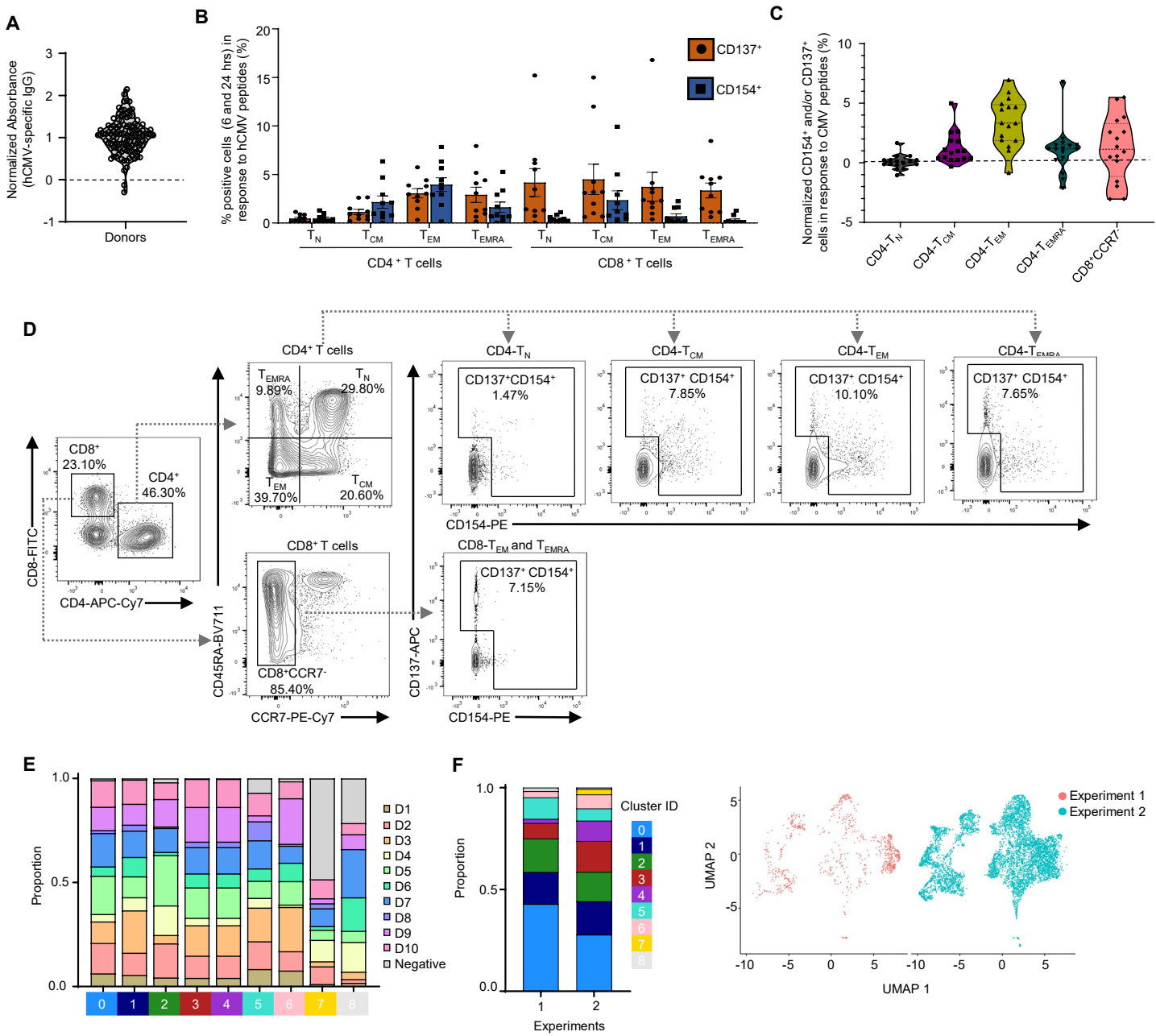

FIGURE S2.

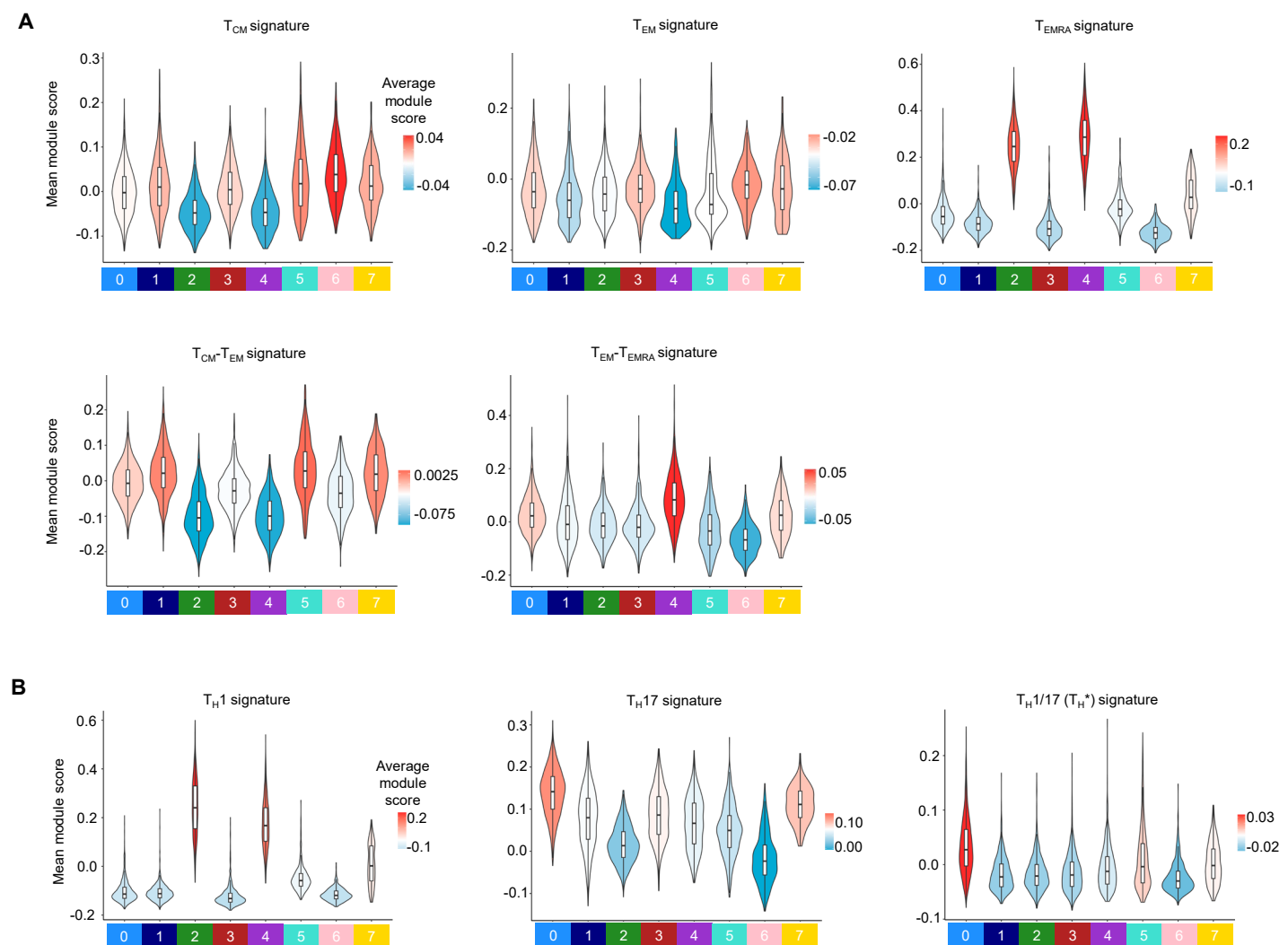

FIGURE S3.

**A**

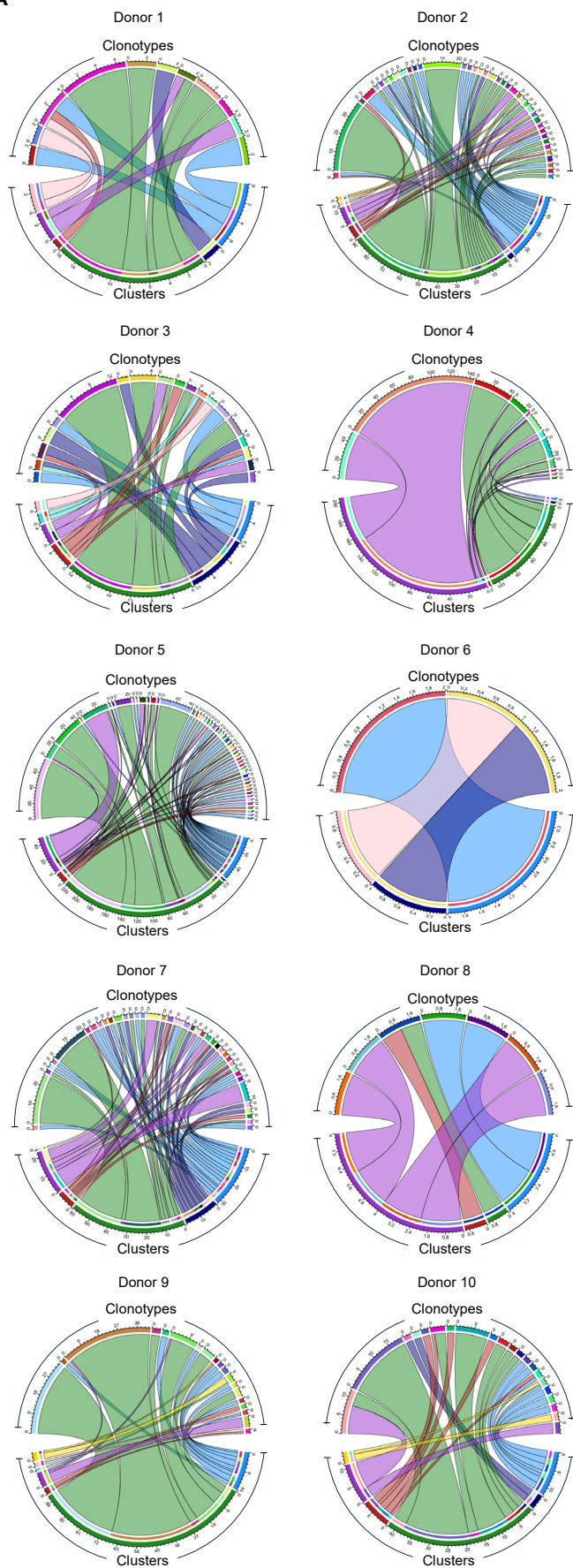

**B**

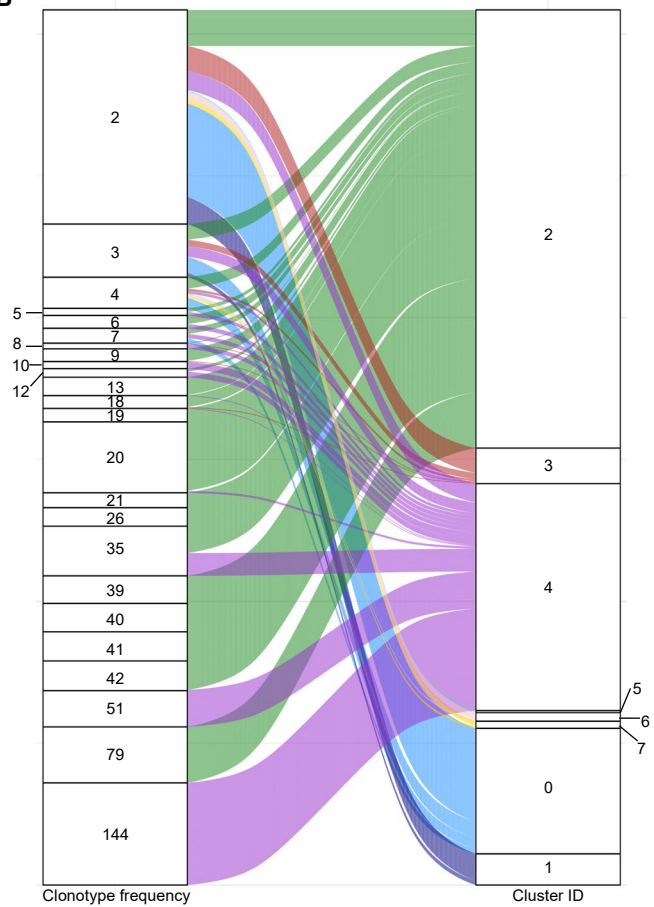

FIGURE S4.

A

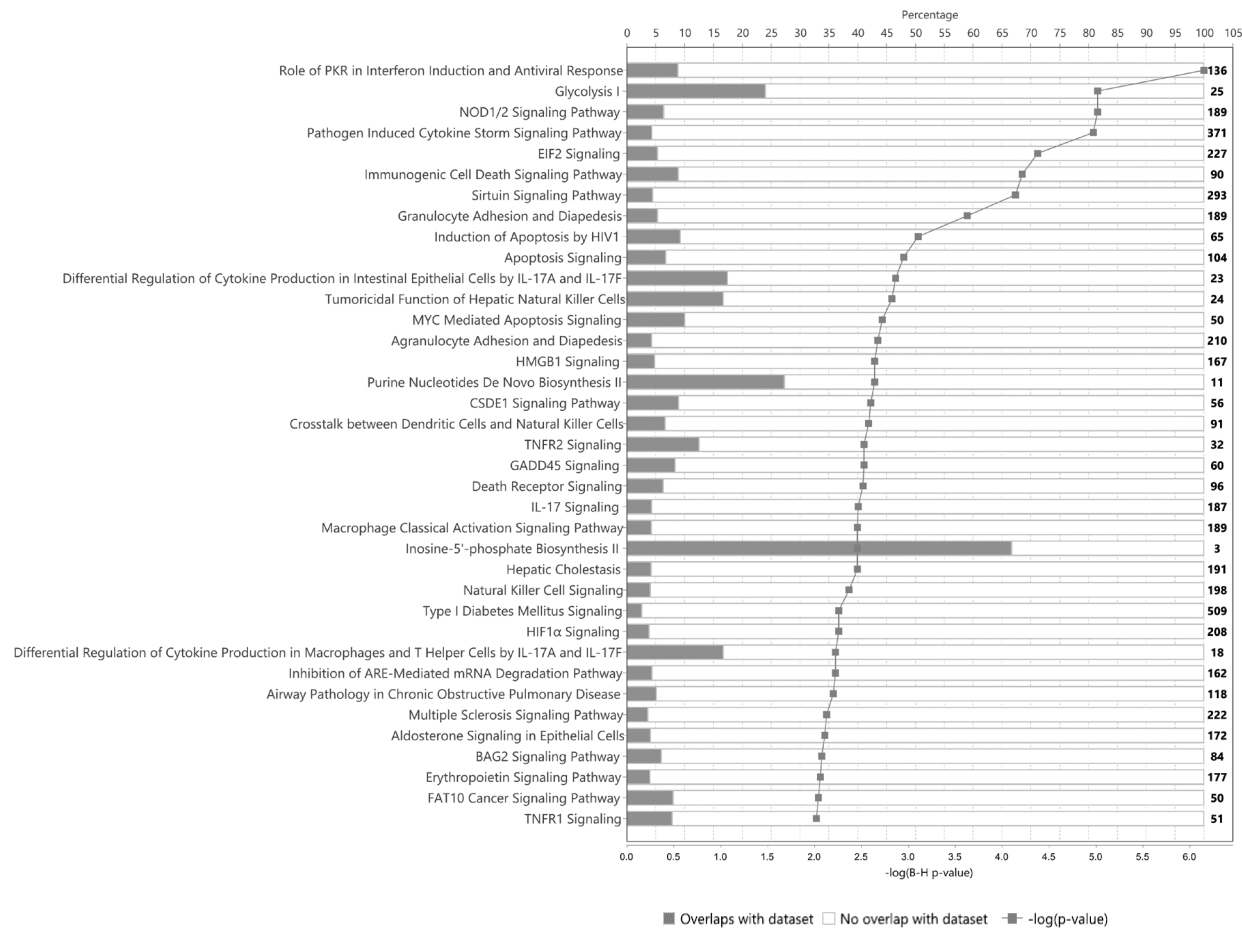

B

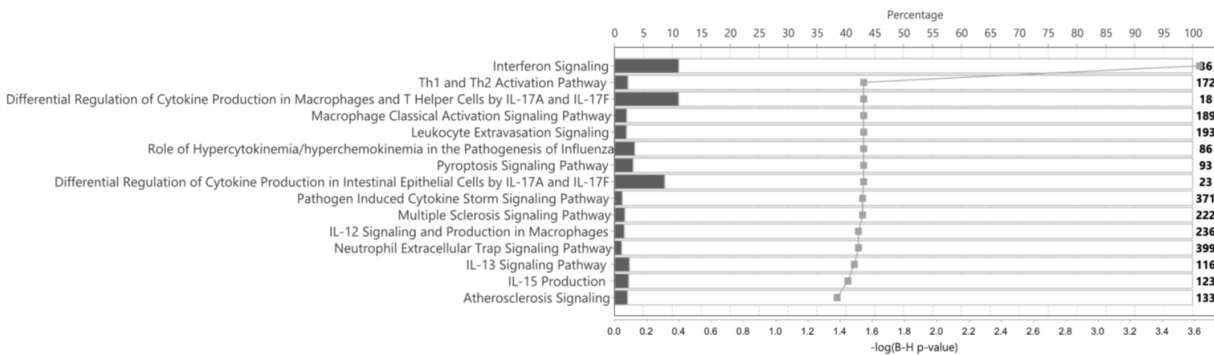

Supplement: Supplementary figures [file EMS194838-supplement-Supplementary_figures.pdf]
